# Effect of CaCl_2_-Induced Surface Gelatinization on Enzymatic Porous Starch

**DOI:** 10.3390/foods14244221

**Published:** 2025-12-09

**Authors:** Nianxia Sun, Yakun Wang, Jie Zhang, Zesheng Wu, Mengting Cheng, Hui Shen, Dianlei Wang, Bo Cui

**Affiliations:** 1 State Key Laboratory of Biobased Material and Green Papermaking, Qilu University of Technology, Shandong Academy of Sciences, Jinan 250353, China; 2 College of Pharmacy, Anhui University of Chinese Medicine, Hefei 230012, China; yakunvv@163.com (Y.W.); 18019594765@163.com (J.Z.); zeshengwu2022@163.com (Z.W.); mc5612@163.com (M.C.); 18155602280@163.com (H.S.); dlwang@ahtcm.edu.cn (D.W.)

**Keywords:** enzymatic porous starch, CaCl_2_ gelatinization, structure characterization, functional property

## Abstract

Porous starch (PS) is widely used in food, pharmaceutical, and environmental industries for its high adsorption capacity and controlled release properties. To explore how surface gelatinization affected enzymatically prepared PS, corn starch was first modified via surface gelatinization using a CaCl_2_ solution and then treated with α-amylase and amyloglucosidase to synthesize PS. Its structural and functional characteristics were subsequently analyzed. The findings demonstrated that the CaCl_2_ solution facilitated the surface gelatinization and enhanced the enzymatic hydrolysis of natural starch. The yield, specific volume, water solubility, swelling power, and oil absorption capacity of PS pretreated with CaCl_2_ solution were improved. After 40 min of processing, the yield, specific volume, and oil absorption capacity of PS reached the optimal state, increasing by 19.90%, 91.19%, and 32.84%, respectively. Consequently, its fisetin encapsulation efficiency (93.67%) and loading capacity (8.03%) were also higher than those of non-pretreated PS, attributed to the reduced short-range structure and crystallinity in the CaCl_2_-pretreated PS. The DPPH and ABTS radical scavenging activities of CaCl_2_-pretreated PS/fisetin (PS/FIT) exceeded those of the non-pretreated PS/FIT and free fisetin. These findings highlight the potential of CaCl_2_ pretreatment as an effective strategy to enhance the functional properties of enzymatic PS.

## 1. Introduction

Starch serves not only as a major carbohydrate source in the human diet but also as a crucial material across the food and pharmaceutical fields. Nevertheless, natural corn starch has intrinsic drawbacks that restrict its extensive use in processing, including low solubility, high viscosity, and easy aging [1,2]. Porous starch (PS) is a modified starch variant featuring reasonably uniform surface pores and is widely used in food, pharmaceutical, and environmental industries. Owing to its unique structure, PS exhibits great adsorption properties and can protect substances that are susceptible to light, oxygen, and temperature, allowing it to be extensively used as an adsorbent, encapsulation agent, and safeguard for sensitive materials [3].

To prepare PS, enzymatic hydrolysis is typically conducted, with the reaction temperature controlled to avoid gelatinization to preserve the starch granular morphology [4]. For producing PS with larger pores, hydrolyzing starch using α-amylase (AM) and amyloglucosidase (AMG) (either in combination or individually) is more effective. AM, an endo-amylase, randomly breaks 1,4-glycosidic bonds in the polysaccharide chain, while AMG, an exo-amylase, acts on the reducing end of 1,4-glycosidic bonds and the non-reducing end of 1,6-glycosidic bonds simultaneously, resulting in the formation of regular pores post-hydrolysis [5,6]. However, native starch is inherently resistant to hydrolysis, primarily due to its densely packed granular structure, double-helical semi-crystalline arrangement, and poor solubility in cold water [7]. Preparation of PS by enzymatic hydrolysis is usually low in efficiency and has poor properties [8,9]. Thus, improving the enzymatic efficiency and the properties of PS is required to expand its processing applicability.

Natural starch predominantly exists as granules, which are divided into four levels: molecules, blocklets, shells, and granules [10,11]. The complete particle structure is composed of alternately arranged shell structures, namely a soft shell and a hard shell. The connection of the soft shell leads to a certain elasticity of the starch particles, and the existence of the hard shell can limit the expansion of particles to a certain extent. Among them, the outermost hard shell (starch shell) is the first barrier for starch particles to resist the external environment, which determines the properties of starch particles to a certain extent [12,13]. Given the unique shell structure of starch granules, it might be highly meaningful to separate these shells via specific methods prior to enzymatic hydrolysis for PS preparation.

Chemical surface gelatinization has been employed to remove the gelatinized granule surface to explore the internal structure of starch granules [14,15]. Gao et al. [16] noted that suitable surface gelatinization could disrupt the starch shell structure, thereby altering its viscoelastic behavior, breaking the hydrogen bonds in the lamellar structure, and reducing the starch crystallinity. Similarly, Zhang et al. [17] found that surface gelatinization-based deshelling of potato starch changed the pasting behavior of starch and improved its solubility and resistant starch levels. Given its low toxicity and cost advantage, CaCl_2_ has emerged as a highly promising gelatinizing agent for starch-based systems. It was reported that starch could be gelatinized in a high concentration of CaCl_2_ solution (about 4 mol/L) at room temperature, and the 4 mol/L CaCl_2_ solution induced starch gelatinization on the surface and decomposed starch granules layer by layer [18]. The use of salts offers distinct chemical environments that can interact with starch chains, affecting starch solubility and gelatinization behavior. Cl^−^ anion can alter the ionic strength of the solution and shield electrostatic forces within the granule, weakening internal hydrogen bonds and facilitating partial solubilization [19]. The Ca^2+^ ions possess a high hydration capacity and charge density. They competitively bind to hydroxyl groups on starch molecular chains as well as to water molecules, thereby disrupting the original hydrogen bonds between starch molecules [20]. Furthermore, surface gelatinization treatments have been shown to disrupt the shell structure, creating more active sites that enhance starch modification efficiency [21]. Drawing on these findings, separating starch shells might not only modify the physicochemical characteristics of starch but also facilitate the penetration of modifiers into starch granules. Nonetheless, limited research has been conducted regarding the effect of CaCl_2_-induced surface gelatinization on the structural and functional properties of enzymatically produced PS.

Therefore, the present study aimed to employ CaCl_2_ to disrupt the starch shell structure, followed by enzymatic treatment, with the objectives of improving the enzymatic efficiency and increasing the solubility of PS as well as elucidating the effects of these modifications on the structural and functional properties of enzymatic PS. This study might be helpful to enhance the functional properties of enzymatic PS using CaCl_2_-induced surface gelatinization in the near future.

## 2. Materials and Methods

### 2.1. Materials

Corn starch (≥98%, 27.1% amylose), α-amylase (AM) (EC 3.2.1.1, 100,000 U/g), and fisetin (>98%) were provided by Macklin Biochemical Co., Ltd. (Shanghai, China). Amyloglucosidase (AMG) (EC 3.2.1.3, 100,000 U/mL) was supplied by Beijing Solarbio Science & Technology Co., Ltd. (Beijing, China). CaCl_2_ (≥97%) was purchased from Aladdin Reagent Co., Ltd. (Shanghai, China). All other reagents utilized herein were distinguished by the analytical-grade quality, barring exceptions otherwise delineated.

### 2.2. Pretreatment of Starch with CaCl_2_ Solution

According to the methodology described by Liang et al. [1], specific modifications were made to the surface gelatinization technique for corn starch. A mixture of native corn starch (20 g) and CaCl_2_ solution (150 mL, 4 mol/L) was continuously agitated in a water bath at 30 °C for 0, 10, 20, 40, 60, and 80 min, respectively, to obtain starch samples with different degrees of gelatinization. The gelatinization process was then terminated by mixing with 1000 mL of pre-cooled ice water at 4 °C. The gelatinized solution was centrifuged at 4000 r/min for 10 min. After discarding the supernatant, the residual CaCl_2_ was completely removed by washing the precipitate three times with 2 L of ultrapure water.

### 2.3. Gelatinization Degree and Enzyme Sensitivity of Starch Treated with CaCl_2_ Solution

The starch treated with different gelatinization times was weighed. The degree of starch gelatinization was computed utilizing the subsequent Formula (1):

(1) $$\mathrm{Gelatinization}\;\mathrm{degree}\;\left(\%\right)=\left(1-\frac{M1}{M2}\right)\times 100\%$$

where M1 represents the retained granule mass (g) and M2 represents the original granule mass (g).

Enzyme sensitivity attributed to intakes previously subjected to CaCl_2_ treatment was quantified by applying the 3,5-dinitrosalicylic acid method [22]. Briefly, 0.4 g of the hulled sample was blended in 25 mL of PBS (pH 4.5). After that, 1 mL of enzyme solution (1 mg of AM, 3 mg of AMG, distilled water to 1 mL) was added, and it was reacted at a constant temperature of 50 °C for 1 h. The reaction was immediately terminated by adding 4% sodium hydroxide. The mixture was centrifuged at 4000 r/min for 10 min. The amount of glucose released from the supernatant was measured using 3,5-dinitrosalicylic acid, and enzyme sensitivity was calculated with the following Formula (2):

(2) $$\mathrm{Enzyme}\;sensitivity\;(\%)=\frac{C\times V\times 0.9}{M}\times 100\%$$

where C represents the glucose concentration (mg/mL), V represents the liquid phase volume (mL) in the reaction system, 0.9 is the conversion coefficient from glucose to starch, and M is the starch mass (mg).

### 2.4. Scanning Electron Microscopy Observation

The morphological properties of the samples were assessed using a scanning electron microscope (SEM, FEI Quattro S Thermo, Brno, Czech). Prior to being coated with a thin layer of gold/palladium, the samples were affixed to an aluminum stub using double-sided tape [23].

### 2.5. Preparation of PS

The procedure for the preparation of PS via complex enzymatic digestion was modeled on the method of Ma et al. [24] with minor modifications. A total of 15 g of starch was dispersed in 100 mL of citric acid–sodium citrate buffer (pH 5.4) and stirred at 42 °C for 10 min. Subsequently, 2% (*w*/*w*) of the complex enzyme, consisting of AM and AMG at a ratio of 1:3, was added to the mixture, which was then incubated with shaking at 42 °C for 20 h [25]. At the end of the reaction, 5 mL of 4% NaOH solution was added, and the resulting suspension was centrifuged at 3000 r/min for 10 min to discard the supernatant. The sediment was washed thoroughly with 2 L of ultrapure water three times. After that, the obtained pellets were collected and dried at 40 °C for 48 h. Finally, the yield of PS was calculated, and its morphological characteristics were observed using SEM. The surface pore area of PS was measured using ImageJ software (Version 1.52a National Institutes of Health, Bethesda, MD, USA).

### 2.6. Specific Volume, Water Solubility, Swelling Power, and Oil Absorption of PS Pretreated with CaCl_2_ Solution

The specific volume of PS was determined as expounded in the methodologies of He et al. [26]. A total of 2 g of PS was allowed to fall freely into a 10 mL measuring cylinder, and the cylinder was gently shaken to ensure the surface of the starch was level. The specific volume was calculated as the volume per gram of starch.

Water solubility, swelling power, and oil absorption capacity were estimated using the method reported by Han et al. [5]. Specifically, an exactitude of 0.4 g of PS underwent dispersion within 20 mL of distilled water. Then, it was heated at 60 °C for 30 min, followed by room-temperature equilibration facilitated via ice water surroundings. After centrifugation at 4000 r/min for 20 min, the sediment and the supernatant showed a clear division into equal parts. Then, the samples were dried until a stable weight was achieved. Water solubility and swelling power of starch were calculated according to Formulae (3) and (4):

(3) $$Solubility\;(\%)=\frac{A}{W}\times 100\%$$

(4) $$Swelling\;power\;(\%)=\frac{B}{W\times (1-S)}\times 100\%$$

where B is the weight of the precipitate (g), W is the weight of PS (g), and A (g) is the mass of the dried supernatant.

The oil absorption capacity of the starch was determined according to Han et al. [5]. Briefly, 1 g of starch was mixed with 10 mL of soybean oil and shaken at 25 °C with a rotating speed of 200 r/min for 60 min to ensure complete oil adsorption. Subsequently, the mixture was centrifuged at 3000 r/min for 15 min. After carefully decanting the upper oil layer, the precipitate was inverted and drained until no oil droplets oozed out. The oil absorption capacity of PS was calculated based on the mass difference before and after adsorption, using the following Formula (5):

(5) $$Oil\;absorption\;(\%)=\frac{W2-W1}{W1}\times 100\%$$

where W1 is the weight of PS (g) and W2 is the PS mass (g) with oil absorbed.

### 2.7. Preparation and Absorption of Fisetin into PS Pretreated with CaCl_2_ Solution

**Scheme 1:**
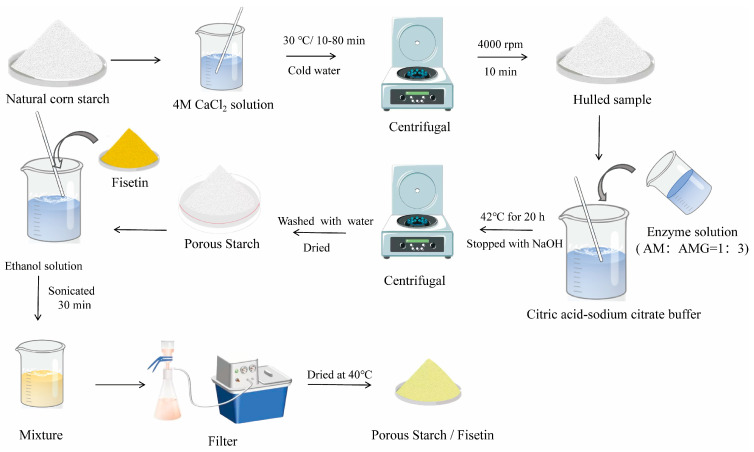
Schematic diagram of the formation of CaCl_2_ pretreated starch, PS, and PS/FIT.

PS-loaded fisetin (PS/FIT) was prepared with slight modifications to the method described by Liu et al. [27], with the assistance of sonication. Initially, 400 mg of PS pretreated with CaCl_2_ solution for 40 min (40PS) and untreated PS (NPS) were mixed with 40 mL of ethanol solution, respectively, and 6 mL of the fisetin solution (4 mg/mL in ethanol) was added to the mixture, which was subsequently sonicated at room temperature for 30 min. The system was then filtered, and the precipitate was dried at 40 °C (details are shown in Scheme 1). Fisetin in the supernatant was determined by UV spectrophotometry at 365 nm to calculate the encapsulation efficiency (EE) and loading capacity (LC) using the following Formulae (6) and (7). Its morphological characteristics were observed using SEM.

(6) $$Encapsulation\;efficiency\;(EE,\%)=\frac{M1-M}{M1}\times 100\%$$

(7) $$Loading\;capacity\;(LC,\%)=\frac{M1-M}{M2}\times 100\%$$

where the content of free fisetin in the supernatant is recorded as M (g), M1 denotes the weight of the total amount of fisetin (g), and M2 (g) denotes the total weight of the precipitate.

### 2.8. Structural Characterizations of CaCl_2_ Pretreated Natural Starch, PS, and PS/FIT

#### 2.8.1. Fourier Transform Infrared Determination

The starch sample (1.5 mg) was mixed with KBr (150 mg), and the Fourier transform infrared (FTIR) spectra were obtained by a Fourier transform infrared spectrometer (Nicolet 6700, Thermo, Waltham, MA, USA) from 4000 to 500 cm^−1^ with a resolution of 4 cm^−1^.

#### 2.8.2. X-Ray Diffraction Analysis

The X-ray diffraction (XRD) patterns of the samples were measured using a Bruker X-ray diffractometer (D8 Advance, Bruker Inc., Karlsruhe, Germany). The scanning rate was 2°/min, with the diffraction angle range set from 5° to 40°, following the procedure described in the previous literature [28]. The instrument operated at a target voltage of 40 kV and a current of 40 mA, with Cu Kα radiation employed. The degree of crystallization (%) was processed by MDI Jade 6.0 software (Materials Data, Inc., Livermore, CA, USA).

#### 2.8.3. Thermal Characterization

The gelatinization properties of the sample and the encapsulation state of fisetin were investigated using differential scanning calorimetry (DSC). A certain amount of starch (3.0 mg) was accurately weighed, added with triple the weight of deionized water, hermetically sealed in an aluminum pan, and equilibrated overnight at room temperature. Subsequently, the thermal behavior of the samples was measured using differential scanning calorimetry (DSC-1 Mettler-Toledo, Columbus, OH, USA). An empty pan was used as a reference. The samples were scanned from 30 to 100 °C at a heating rate of 10 °C/min under a nitrogen atmosphere. The gelatinization temperatures of onset temperature (T_o_), peak temperature (T_p_), conclusion temperature (T_c_), and enthalpy ΔH were recorded. Under a nitrogen atmosphere, pure fisetin and the inclusion complex were heated from 50 °C to 400 °C at a programmed rate of 10 °C/min. The encapsulation state was evaluated by monitoring the changes in the characteristic melting peak of pure fisetin within the inclusion complex.

### 2.9. Antioxidant Activity of CaCl_2_ Pretreated PS/FIT

The antioxidant capacities of free fisetin and PS/FIT were assayed using the DPPH radical scavenging assay and the ABTS free radical scavenging kit. Briefly, sample solutions of varying concentrations were prepared by dissolving free fisetin (1 mg/L, 2 mg/L, 3 mg/L, 4 mg/L), NPS/FIT, and 40PS/FIT (containing the same fisetin content as the free fisetin solutions) in anhydrous ethanol. For the ABTS assay, the ABTS solution was diluted 50-fold with PBS to prepare the working solution. Subsequently, 200 μL of ABTS solution was transferred into each well of a microplate, followed by the addition of 10 μL of sample solution to the corresponding wells. The reaction was performed in the dark, and after 6 min, the absorbance was measured at 405 nm. For the DPPH assay, 3 mL of DPPH solution (1 × 10^−4^ mol/L) was mixed with 1 mL of fisetin or PS/FIT solutions of varying concentrations. The mixture was incubated at room temperature for 30 min in the dark, after which, the absorbance was measured at 517 nm using a UV spectrophotometer (UVmini-1285, SHIMADZU, Kyoto, Japan). The DPPH radical scavenging capacity was calculated using the following Formula (8):

(8) $${SA}_{DPPH}(\%)=[1-\frac{\left(A2-A1\right)}{A0}]\times 100\%$$

where A0 is the absorbance of the solution without the sample, A1 is the background value of the added sample, and A2 is the absorbance of the added sample.

### 2.10. Statistical Analysis

Analysis of variance (ANOVA) was conducted using SPSS Statistics software (Version 22, SPSS Inc., Chicago, IL, USA) to analyze the differences among treatment groups. Differences were considered statistically significant when *p* < 0.05. All experiments were conducted at least in triplicate, and the data were reported as mean ± standard deviation. In addition, principal component analysis (PCA) and correlation heatmap were performed using GraphPad Prism software (Version 9.5, GraphPad Software, San Diego, CA, USA).

## 3. Results and Discussion

### 3.1. Effect of CaCl_2_ Soaking Time on Gelatinization Degree and Enzyme Sensitivity of Starch

**Figure 1:**
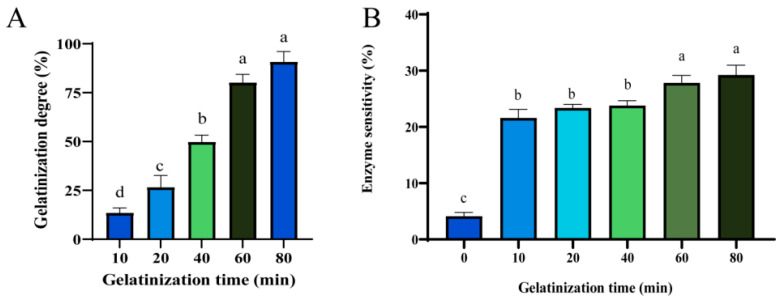
Effect of CaCl_2_ soaking time on gelatinization degree (**A**) and enzyme sensitivity (**B**) of starch. Different treatment groups with different letters were significantly different (*p* < 0.05).

It was reported that starch could be gelatinized in a high concentration of CaCl_2_ solution (about 4 mol/L) at room temperature, and a 4 mol/L CaCl_2_ solution induced starch gelatinization on the surface and decomposed starch granules layer by layer [18]. In this study, the raw corn starch was pretreated by the CaCl_2_ solution for varying durations to obtain hulled starch samples with different treatment degrees. As shown in Figure 1, both the gelatinization degree (A) and enzyme sensitivity (B) of starch increased rapidly with prolonged CaCl_2_ treatment time. It was revealed that the starch granules’ outer shell structure was disturbed and gelatinization was induced at their periphery by the CaCl_2_ solution [16,29]. This erosive activity caused the starch granule structure to become rougher and fragmented as the gelatinization period rose, thereby resulting in a higher gelatinization degree with longer treatment duration (Figure 1A). The gelatinization degree curve was further analyzed, and the correlation coefficient (R^2^) was used to select the model that best described the formulation. According to the fitting result of the kinetics, the changes in the gelatinization degree of starch over time could be fitted by the first-order kinetic model, and the fitting degree (R^2^ = 0.9681) was relatively good, indicating that, within the experimental time range, the variation in gelatinization degree with time had a good regularity. The linearization kinetics equation of the gelatinization degree had a slow slope (0.0326 min^−1^), suggesting that the gelatinization process proceeded relatively slowly. The induction period t_0_, calculated based on the fitting parameters, was 10.41 min, and the half reaction time t_1_/_2_ was 31.68 min, indicating that it took a relatively long time for the sample to reduce the gelatinization degree by half from the start of treatment, and it had certain structural stability.

The ratio of amylose to amylopectin, crystalline structure, and size of the particles all had an impact on enzyme sensitivity [30]. Cations with larger ionic radii (Ca^2+^) could improve the protonation of anions. Protonation of anions could weaken the hydrogen bonds between starch molecular chains, thereby promoting the rupture of granules and the surface gelatinization. The starch shell’s edge might contain the majority of the amylose in starch, forming a stable and orderly crystalline structure [1]. The orderly arrangement of starch granules was disturbed when CaCl_2_ broke down the outer shell structure of starch. Given that starch granules’ amorphous areas were more vulnerable to AM and AMG deterioration compared to crystalline regions, surface gelatinization treatment enhanced the accessibility of enzymes to these amorphous areas and increased the enzyme sensitivity (Figure 1B). Correspondingly, the changes in the enzyme sensitivity of the starch sample over time could also be fitted by the first-order kinetic model. The linearization kinetics equation of the enzyme sensitivity had a goodness of fit (R^2^ = 0.903), a slow slope (0.029 min^−1^), and an early t_1_/_2_ (5.08 min), implying that starch had undergone rapid structural changes at the initial stage of enzymatic hydrolysis, and the enzyme sensitivity increased relatively quickly. However, there was no significant difference in the enzyme sensitivities of PS with 10–40 min CaCl_2_ treatment. As the treatment time with CaCl_2_ solution increased (10–40 min), the addition of Ca^2+^ increased the ionic bonds between the starch molecules and cations. Positively charged cations could pair with the separation charge on the starch hydroxyl oxygen atom, leading to crosslinking between starch chains, resulting in the chain recombination of amylose and amylopectin and reducing the mobility of chain segments. Therefore, the crosslinking strengthened the crystalline structure of starch granules, making fewer starch molecules leach.

### 3.2. SEM of Surface Gelatinization Starch

**Figure 2:**
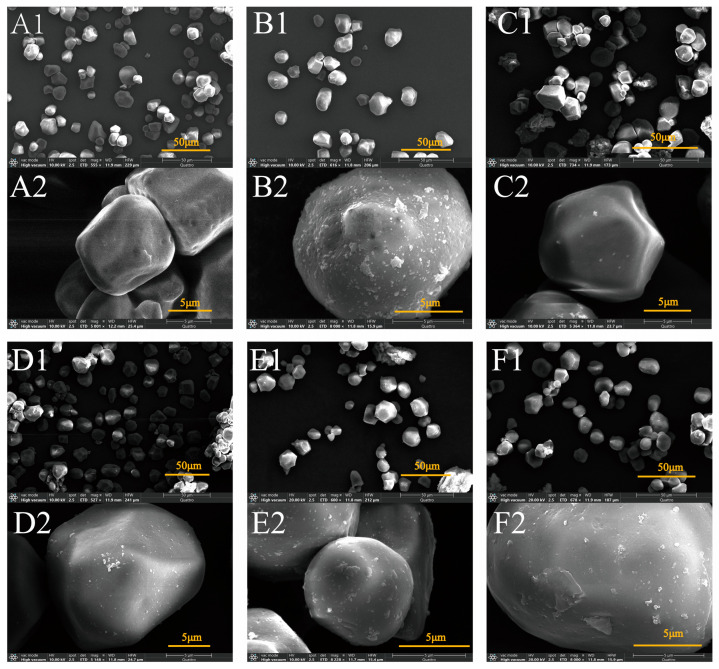
SEM (500× and 5000×) images of native and surface gelatinizated corn starch treated with varying durations. (**A**) native starch; (**B**–**F**) surface gelatinization starch treated with 10 min, 20 min, 40 min, 60 min, and 80 min, respectively. Images (**A1**–**F1**) show the morphology at 500× magnification; images (**A2**–**F2**) show the morphology at 5000× magnification.

Morphological observation serves as a crucial index for evaluating the degree of starch surface gelatinization, offering a more straightforward insight into the impacts of surface gelatinization treatment on them [31]. The morphology of the samples could directly influence their other properties, including bulk density, rehydration characteristics, and the release of the core materials [32]. As shown in Figure 2A, native corn starch granules had spherical or irregular shapes with varying sizes and relatively smooth surfaces, and the result was consistent with the previous study [33]. After surface gelatinization treatment, the starch granules’ overall structural integrity was preserved; however, their edge structures became rough and fractured, while the overall structural integrity of the starch granules was still maintained (Figure 2B–F), which was consistent with previous findings [1]. The breakdown of the starch shell was exacerbated by the hydrolysis of the starch granules’ surface structure by the CaCl_2_ solution. With the increase in surface gelatinization degree, the corners of polygonal starch granules gradually disappeared, and their shapes tended to become more spherical. As the CaCl_2_ treatment time increased (60 min and 80 min), some starch granules underwent disintegration and gelatinization, and this phenomenon became more pronounced with the deepening of the gelatinization degree (Figure 2E,F). Additionally, starch molecules showed signs of adhesion to each other. It had been revealed that, with prolonged treatment with CaCl_2_, the crosslinking formed between Ca^2+^ ions and hydroxyl groups in starch would gradually reach saturation and then might allow water to penetrate into the starch granules, causing them to absorb water, swell, and eventually rupture [14]. Thus, the morphology changes in starch treated with the CaCl_2_ solution provided an intuitive understanding of the effects of surface gelatinization treatment on the gelatinization degree and enzyme sensitivity of starch (Figure 1).

### 3.3. SEM of PS

**Figure 3:**
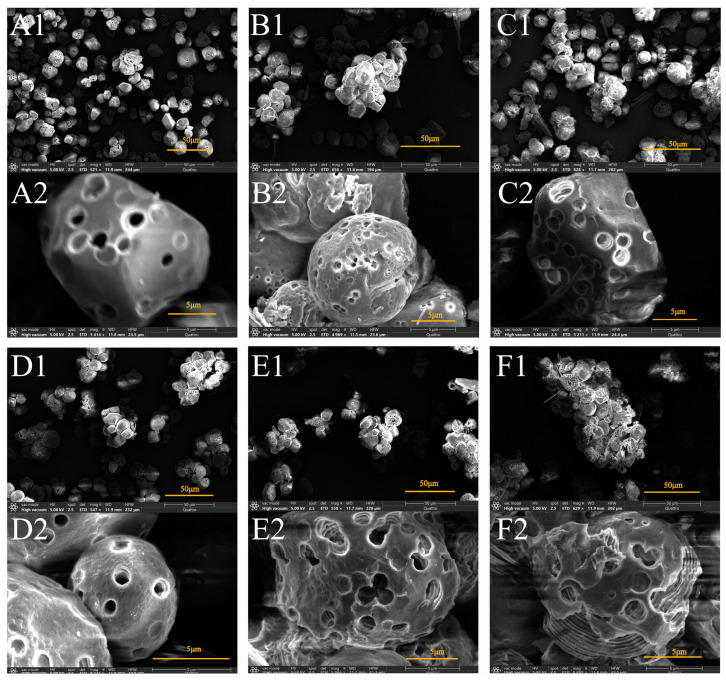
SEM (500× and 5000×) images of PS with/without CaCl_2_ treatment. (**A**) native PS; (**B**–**F**) surface gelatinization PS treated with 10 min, 20 min, 40 min, 60 min, and 80 min, respectively. Images (**A1**–**F1**) show the morphology at 500× magnification; images (**A2**–**F2**) show the morphology at 5000× magnification.

The micrographs of PS with and without CaCl_2_ pretreatment are displayed in Figure 3. As observed in Figure 3A, the surface of PS (without CaCl_2_ pretreatment) was covered with numerous small and dense pores. They were formed by the combined hydrolytic action of AM and AMG, which rapidly broke down glycosidic bonds in starch [3]. These pores varied in depth, with some extending into the interior of the particles, significantly increasing the starch’s specific surface area. For PS samples pretreated with CaCl_2_ (Figure 3B–F), surface gelatinization of the starch granules resulted in a rough and rounded surface. At the same time, the porous structure was still retained, which was similar to the prior research [34]. Notably, PS pretreated with CaCl_2_ exhibited more open pores compared to untreated PS, likely due to reactive erosion of the starch surface. The pore area increased from approximately 1.24 μm^2^ for the untreated starch to 1.53, 1.80, 2.11, 3.34, and 5.78 μm^2^ after 10, 20, 40, 60, and 80 min of CaCl_2_ treatment, respectively. These observations aligned with the trends in starch enzyme sensitivity (Figure 1B). As the pretreatment time with CaCl_2_ was extended, the surface of PS granules developed scalloped edges, existing pores expanded in size, and deeper penetration into the interior of starch granules was observed (Figure 3E,F). Similar morphological trends had been observed in the previous studies [35].

### 3.4. Effect of CaCl_2_ Soaking Time on Yield, Specific Volume, Solubility, Swelling Power, and Oil Absorption of PS

**Figure 4:**
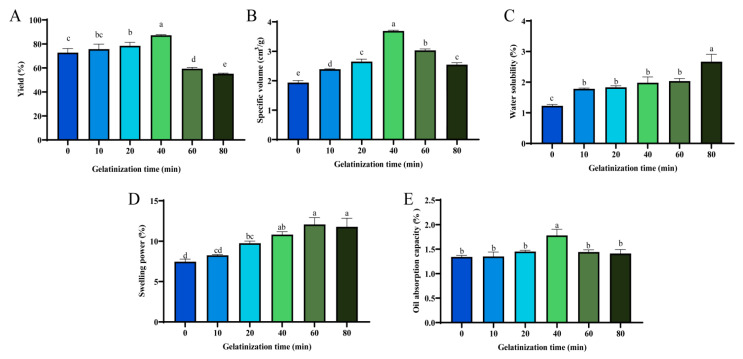
Yield (**A**), specific volume (**B**), water solubility (**C**), swelling power (**D**), and oil absorption capacity (**E**) of PS with/without CaCl_2_ treatment. Different treatment groups with different letters were significantly different (*p* < 0.05).

Corn starch with and without CaCl_2_ pretreatment was subjected to enzymatic hydrolysis to prepare PS, and the yields and specific volume of the resulting PS were further analyzed. As shown in Figure 4A,B, both the yield and specific volume of PS first increased and then decreased with the extension of CaCl_2_ soaking time. These changing trends could be explained by the SEM observations of starch and PS treated with CaCl_2_ solution (Figure 2 and Figure 3). As revealed by the results of SEM of natural starch treated with CaCl_2_ solution (Figure 2), the shell structure of the starch granules was disrupted. This disruption enhanced the accessibility of enzymes to the starch molecules, facilitating more efficient enzymatic hydrolysis in the initial stage, thus leading to an increase in PS yield as the CaCl_2_ soaking time was prolonged. However, when the CaCl_2_ treatment time was further extended, the surfaces of the PS granules generated scalloped edges (Figure 3), and excessive degradation of the starch granules occurred, resulting in a subsequent decrease in yield. As shown in the result of SEM of PS, numerous small pores and channels gradually formed inside the particles during the initial stage of gelatinization (10–40 min), which increased the specific volume. As gelatinization progressed further, aggregation and adhesion between starch granules became evident, partially filling or destroying the previously formed pore structures and leading to a decrease in the specific volume of PS.

To further characterize the porosity-related properties of PS pretreated with CaCl_2_ solution, its water solubility, swelling power, and oil absorption capacity were analyzed, with the results presented in Figure 4C–E. The water solubility and swelling power of PS were influenced by granule integrity and starch chain interactions [36]. PS treated with CaCl_2_ solution exhibited higher water solubility compared to the untreated control (Figure 4C). This phenomenon was associated with the surface gelatinization induced by CaCl_2_, which weakened the structural strength of the starch sample. The reduced intermolecular interactions between starch molecules promoted the leaching of soluble components into the aqueous phase, thereby increasing the water solubility of PS treated with CaCl_2_ solution. The swelling power of PS initially increased with prolonged CaCl_2_ soaking time (Figure 4D). This could be attributed to the increased number of pores and the disruption of amorphous regions in the starch granules during the early stages of treatment [37,38], which facilitated the water absorption and swelling power. Figure 4E shows that surface gelatinization of PS treated for 40 min had a higher oil absorption capacity than the control. Sequential AM and AMG hydrolysis produced abundant mesopores on the starch granules pretreated with CaCl_2_, and the oil absorption accordingly increased [39]. The results were consistent with the micrographs of PS with/without CaCl_2_ treatment (Figure 3).

**Figure 5:**
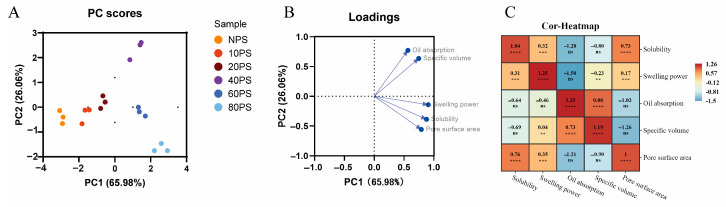
The score map (**A**), loading map (**B**), and correlation analysis heatmap (**C**) of structural–physicochemical properties of PS. Note: **, ***, **** indicate significant diferences at the 0.01, and 0.001 and 0.0001 levels, respectively; ns indicates no significant difference (*p* > 0.05).

Furthermore, PCA and correlation analysis were used to correlate the physicochemical properties and structural properties, and the results are shown in Figure 5. In Figure 5A, PC1’s (65.98%) and PC2’s (26.06%) combined contributions totaled 92.05%, implying highly effective for subsequent analysis. As the CaCl_2_ gelatinization time increased, the coordinates of the samples shifted toward the positive half-axes of the X-axis, and only PS with 40 min of CaCl_2_ pretreatment distinctly separated from the others and shifted toward the positive half-axes of the Y-axis. Among them, PS with 40 min of CaCl_2_ pretreatment was distinctly separated from others and exhibited different physicochemical properties. The loading plot analysis (Figure 5B) indicated that oil absorption capacity and specific volume of starch were located in the positive half-axis of the Y-axis and had negative correlations with the pore area. Instead, swelling power and solubility were located in the negative half-axis of the Y-axis and had positively correlated with the pore area. The results suggested that the increased pore area increased its swelling power and solubility and inhibited its oil absorption capacity and specific volume. Furthermore, the results of the correlation heatmap (Figure 5C) further supported the aforementioned PCA findings and the hypothesis that pore formation governed the properties. Therefore, considering all factors comprehensively, PS with 40 min of CaCl_2_ pretreatment exhibited the best physicochemical properties.

### 3.5. EE and LC of Fisetin in PS Pretreated with CaCl_2_ Solution

**Figure 6:**
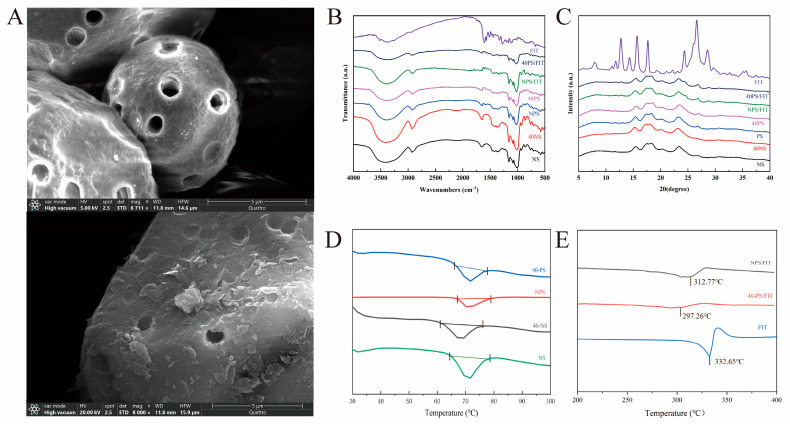
Structural characterizations of CaCl_2_-pretreated nature starch, PS, and PS/FIT. (**A**) Morphological comparison of PS before and after fisetin loading; (**B**) FTIR spectra of NS, PS, FIT, and PS/FIT; (**C**) XRD patterns of NS, PS, FIT, and PS/FIT; (**D**) DSC profiles after gelatinization and enzymatic hydrolysis; (**E**) DSC curves of fisetin and PS/FIT.

Fisetin, a natural component abundant in fruits and vegetables, is endowed with multiple distinct biological properties, including antioxidant ability, anti-angiogenic activity, and so on [40]. However, the applications of fisetin have been hampered due to its poor aqueous solubility and low oral bioavailability [41]. Therefore, following the successful preparation of PS pretreated with CaCl_2_ solution, it was applied to encapsulate fisetin. The EE and LC of fisetin were subsequently measured, in which the EE and LC of 40PS/FIT (93.67 ± 0.89% and 8.03 ± 0.06%) were significantly higher than those of NPS/FIT (54.23 ± 1.66% and 4.15 ± 0.12%). This improvement could be derived from the enhanced open pores and larger specific surface area of 40PS (Figure 3), which endow it with stronger adhesion and adsorption capacities. These pores provide more available binding sites for fisetin molecules, thereby resulting in higher EE and LC. The SEM images illustrated the external surface of 40PS and 40PS/FIT. As shown in Figure 6A, the pores on the outer surface of PS were not easily identified after the encapsulation. In general, solutions could enter into a void of the matrix more easily when the pore diameter was sufficiently big, but the large pore size of encapsulating materials also promoted the leakage of entrapped compounds. The disappearance of apparent pores on 40PS after encapsulation, therefore, might contribute to the protection of encapsulated fisetin during storage and digestion.

### 3.6. Structural Characterizations of CaCl_2_-Pretreated Nature Starch, PS, and PS/FIT

#### 3.6.1. FTIR Analysis

Figure 6B presents the FTIR spectra of the samples, displaying characteristic peaks typical of natural starch. A broad band observed around 3400 cm^−1^ was attributed to O–H stretching vibrations originating from amylose or amylopectin constituents (glucose ring). Sharp peaks observed at 2935 cm^−1^ and 1650 cm^−1^ were associated with asymmetric –C–H stretching and –H–O–H bending vibrations, respectively. Among them, the peak at 2935 cm^−1^ corresponded to C–H stretching vibration of methyl and methylene groups, while that at 1650 cm^−1^ originated from tightly bound water located primarily in the noncrystalline region of starch granules [42]. The bands at 1047 cm^−1^ and 1022 cm^−1^ were indicative of interactions involving C–O–H and –CH_2_ groups, respectively, which were closely linked to starch’s amorphous and crystalline structures [43]. As illustrated in Figure 6B, both the surface gelatinization and enzymatic treatment maintained the intrinsic FTIR profile of starch, suggesting that no new chemical bonds or groups were introduced into starch molecules. Given that surface gelatinization exerted its effect mainly on the starch surface shell, it failed to bring additional chemical bonds or distinctive spectral features in the starch molecule [17,44].

The ratio of R1047/1024 was commonly used to evaluate the short-range structure order in starch [45]. As listed in Table 1 of this study, surface-gelatinized starch (40NS) exhibited a slightly increased R1047/1024 ratio relative to native starch, suggesting that the short-range ordered structure was maintained and the crystalline regions were better organized after the treatment. The result was similar to the reported R1047/1024 ratio of CaCl_2_-treated starch in the previous study [14]. It had been reported that surface gelatinization caused the breakdown of the granular shell, which in turn enhanced the molecular disorder within the starch matrix. As surface gelatinization progressed, the treatment induced fragmentation of granules. Hydrolysis of amorphous regions facilitated the generation of short starch chains, which might polymerize and accumulate in the crystalline region. This process enhanced the stability of the structure, as evidenced by an increase in crystallinity [46,47]. Additionally, interactions between Ca^2+^ and starch molecules might lead to a rise in such molecular disorder [16,17]. The addition of Ca^2+^ could pair with the separation charge on the starch hydroxyl oxygen atom, leading to crosslinking between starch chains, promoting the chain recombination of amylose and amylopectin and increasing short-range ordered structure. The R1047/1024 value of PS was also higher than that of native starch, which aligned with the previous study [36]. Generally, the crystalline regions were more resistant to enzymatic hydrolysis than amorphous regions, with enzymes primarily attacking the latter [48]. However, the ratio of R1047/1024 of PS pretreated with surface gelatinization was lower than that of untreated PS. This might be attributed to the CaCl_2_ pretreatment, which slightly disrupted granular integrity and crystalline structure, thereby increasing the susceptibility of starch to enzyme attack.

The fisetin spectrum exhibited characteristic absorption bands at distinct wavenumbers: 3365 cm^−1^ (O-H stretching from aromatic hydroxyl groups), 1607 cm^−1^ (C=O stretching), 1570 cm^−1^ (C=C stretching), 1447 cm^−1^ (C-O stretching), and 1272 cm^−1^ (C-O-H bending). It was consistent with the previous study [49]. Notably, the peaks of fisetin were absent in the spectra of the PS/FIT. This absence indicated that fisetin was largely entrapped within the matrix of PS [50]. Additionally, no additional peaks emerged in the PS/FIT spectrum, which signified the absence of covalent bonding between fisetin and the PS wall materials. Instead, non-covalent interactions or physical adsorption served as the mediators for the interaction between fisetin and PS [51].

#### 3.6.2. XRD Analysis

The molecular chains of amylose and amylopectin undergo self-assembly to form a multi-scale structure, and these systems exert a direct influence on the physicochemical properties of starch [52]. To assess the crystallinity of the samples, XRD was conducted, and the XRD patterns of NS, PS, fisetin, and PS/FIT are shown in Figure 6C. As a semi-crystalline biopolymer, native starch exhibited a single diffraction peak at 15°, a shoulder peak at 18°, and a strong peak at 23°, which indicated that it had an A-type crystalline configuration. The result was consistent with the previous study [53]. Neither surface gelatinization nor enzymatic treatment caused changes in the starch XRD pattern and retained the crystalline patterns of starch.

The peak intensities in the XRD profile served as an indicator of the crystallinity of the starch granule [54]. To assess changes in crystallinity degree after surface gelatinization and enzymatic treatments, the relative crystallinity of each sample was calculated and is shown in Table 1. Native starch granules generally exhibited a crystallinity ranging from 15% to 45% [55]. Native corn starch had a crystallinity of 32.16%, whereas it slightly decreased to 30.53% after CaCl_2_ pretreatment (Table 1). The result indicated that the disruption of the granule surface broke the hydrogen bonds among hydroxyl groups in the amylopectin molecular chains. The crystallinity of PS slightly increased, and the results were consistent with the results of the previous study [36]. This rise could be attributed to the fact that crystalline regions were more hydrolysis resistant. Enzymatic hydrolysis of raw starch, however, preferentially targeted the amorphous regions, thereby increasing the relative proportion of the crystalline region [48], thereby allowing an increase in the R1047/1024 value (Figure 6B), swelling power, and solubility. After surface gelatinization treatment, the crystallinity of PS decreased. Surface gelatinization of starch granules disrupted their shell structure, disturbing the internal ordering of starch molecules, as supported by FTIR evidence of diminished short-range structures. This disruption might contribute to the improved ability of PS pretreated with CaCl_2_ to encapsulate fisetin.

The fisetin diffractogram exhibited distinct sharp diffraction peaks at 2θ angles 12.5, 15.5, 17.5, 26.6, and 28.5, confirming its crystalline nature [56]. These characteristic sharp peaks of fisetin were significantly reduced in intensity or entirely absent in the diffractogram of PS/FIT, indicating that fisetin existed within the complex, which could be attributed to the encapsulation of the fisetin into the PS matrix.

#### 3.6.3. Thermal Properties

The thermal properties of NS, 40NS, NPS, 40PS, NPS/FIT, 40PF/FIT, and fisetin were measured by DSC. As is shown in Figure 6D, all samples depicted a single endothermic peak. Furthermore, starch’s T_o_, T_p_, and T_c_ were reduced after surface gelatinization treatment. This phenomenon was primarily attributed to the destruction of starch by the CaCl_2_ solution during surface gelatinization. This could allow water molecules to enter starch more efficiently, thus lowering the initial pasting temperature of starch. Meanwhile, CaCl_2_ solution could compete for and disrupt the hydrogen bonding network that maintained the structural stability of starch granules, thereby lowering the energy required for the gelatinization process [57]. The T_o_, T_p_, and T_c_ temperatures for NPS were recorded as 66.93, 70.19, and 78.62 °C, respectively. Surface gelatinization treatment led to a decrease in the gelatinization temperature of PS. This might be attributed to the CaCl_2_ pretreatment, which slightly disrupted granular integrity and crystalline structure, thereby increasing the susceptibility of starch to enzyme attack and lowering the gelatinization temperature of PS. In addition, the enthalpy of starch showed a decreasing trend with the surface gelatinization treatment (Table 1). The enthalpy reflected the energy required for heat absorption during the starch phase transition, which was accompanied by the crystalline-to-liquid transformation of the structure. Those findings were generally consistent with our previous SEM and other structural characteristic results.

The melting behavior of fisetin was characterized by a sharp endothermic peak at 332.66 °C (Figure 6E), corresponding to its melting or decomposition. The crystalline structure of fisetin was relatively stable, requiring a higher temperature to disrupt its crystal lattice for melting or decomposition. In the NPS/FIT sample, the melting peak appeared at 312.77 °C. This phenomenon could be attributed to the encapsulation of fisetin within PS. The progressive weakening of the characteristic fisetin peak in 40PS/FIT indicated a significant reduction in crystallinity. Compared to NPS/FIT, the fisetin in 40PS/FIT was more effectively encapsulated. This confirmed the successful transition from a crystalline state to an amorphous form dispersed within the starch carrier.

### 3.7. Antioxidant Activity

**Figure 7:**
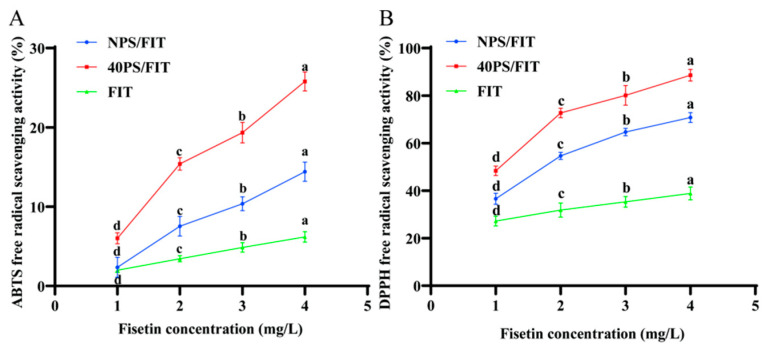
ABTS (**A**) and DPPH (**B**) scavenging activities of free fisetin, NPS/FIT, and 40PS/FIT. The data are expressed as mean ± standard deviation (n = 3). Different letters indicate statistical significance (*p* < 0.05).

Fisetin had an excellent activity in scavenging various radicals. In this research, the antioxidant capacities of free fisetin and PS/FIT pretreated with/without CaCl_2_ were evaluated using DPPH and ABTS free radical scavenging assays, and higher radical scavenging activity corresponded to better antioxidant activity (Figure 7). DPPH is a stable radical widely used to evaluate antioxidant activity. When antioxidants like fisetin are present, DPPH is reduced, accompanied by a color change from purple to yellow (the color of diphenyl picryl hydrazine). This process causes a decrease in absorbance and an increase in scavenging percentage [58]. Similarly, the ABTS assay measures the antioxidant activity of biological compounds. When ABTS radicals encounter a proton-donating substance, they are scavenged, which in turn reduces absorbance. The scavenging percentage is determined by the fading of the blue-green color of ABTS following the addition of the antioxidant as well as the corresponding decrease in its absorbance value [59].

As shown in Figure 7, when the concentration of fisetin increased, its DPPH and ABTS radical scavenging activities gradually increased. Fisetin had a good DPPH and ABTS radical scavenging abilities, with IC50 values of 13.64 ± 1.42 mg/L and 94.35 ± 2.56 mg/L, respectively. Notably, the DPPH and ABTS radical scavenging activities of PS/FIT pretreated with CaCl_2_ (IC50 = 1.64 ± 0.04 mg/L and 16.97 ± 0.75 mg/L, respectively) and PS/FIT pretreated without CaCl_2_ (IC50 = 1.04 ± 0.02 mg/L and 10.53 ± 0.35 mg/L, respectively) were higher than those of the free fisetin. From the results acquired, encapsulating fisetin did not suppress its radical scavenging activity; instead, it notably boosted its antioxidant activity. Combined with the results of the solubility of PS and the synergistic effect between PS and fisetin, the hydrophobic property of fisetin might be regulated by the hydrophilic pores of PS, thereby improving the solubility of 40PS/FIT. This same phenomenon was documented in a prior study [60]. Regarding PS/FIT and 40PS/FIT, their relatively higher scavenging activity was probably a result of 40PS/FIT possessing better solubility and stronger antioxidant activity than NPS/FIT, in addition to variations in the carrier materials. Therefore, CaCl_2_ pretreatment as an effective strategy might enhance the functional properties of enzymatic PS to increase the antioxidant activity of bioactive compounds.

## 4. Conclusions

In this study, the effect of CaCl_2_-induced surface gelatinization treatment on the structure-property improvement of PS was elucidated. Surface gelatinization treatment could break up the surface structure, disrupt the crystal structure, and interfere with the short-range ordered structure of PS because the CaCl_2_ solution could disturb the outer shell structure of starch and calcium ions could form a crosslinked structure in granules through electrostatic interactions with the hydroxyl group of starch, thereby increasing the physicochemical and functional properties. Therefore, CaCl_2_-induced surface gelatinization treatment as a potential pretreatment could enhance the structural and functional properties of PS, thus expanding its in-depth applications in food and pharmaceutical fields. However, there are still many limitations, such as a lack of in vivo antioxidant validation, the scalability of 4 M CaCl_2_ use, and the release profile of fisetin from PS. Future attention should also be focused on using this study’s findings as a basis for other bioactives, release kinetics of bioactive components, and the integration of the food system.

## Figures and Tables

**Table 1:** The FTIR patterns, XRD characteristics, and DSC patterns of native starch, PS, fisetin, and PS/FIT.

Sample	FTIR	XRD		DSC			
	1047/1022 cm^−1^	Type	RC (%)	T_o_ (°C)	T_p_ (°C)	T_c_ (°C)	ΔH_gel_ (J/g)
NS	1.054 ± 0.004 ^c^	A	32.16 ± 0.05 ^d^	64.80 ± 0.32 ^b^	71.68 ± 0.38 ^a^	76.29 ± 0.46 ^b^	1.83 ± 0.04 ^a^
40NS	1.103 ± 0.004 ^a^	A	30.53 ± 0.05 ^f^	62.42 ± 0.41 ^c^	69.33 ± 0.20 ^c^	73.94 ± 0.67 ^c^	0.46 ± 0.03 ^b^
NPS	1.085 ± 0.007 ^b^	A	33.05 ± 0.05 ^c^	66.93 ± 0.38 ^a^	70.19 ± 0.27 ^b^	78.63 ± 0.33 ^a^	0.22 ± 0.02 ^c^
40PS	1.046 ± 0.002 ^d^	A	31.80 ± 0.05 ^e^	67.11 ± 0.22 ^a^	71.68 ± 0.26 ^a^	76.29 ± 0.35 ^b^	0.16 ± 0.02 ^d^
NPS/FIT	1.060 ± 0.006 ^c^	A	35.00 ± 0.05 ^b^	/	/	/	/
40PS/FIT	1.044 ± 0.003 ^d^	A	35.00 ± 0.05 ^b^	/	/	/	/
FIT	1.033 ± 0.007 ^e^	/	74.01 ± 0.05 ^a^	/	/	/	/

The data are expressed as mean ± standard deviation (n = 3). Different letters in each column indicate statistical significance (*p* < 0.05).

## Data Availability

The original contributions presented in this study are included in the article. Further inquiries can be directed to the corresponding authors.

## Abbreviations

The following abbreviations are used in this manuscript:

PS	porous starch
AMG	amyloglucosidase
AM	α-amylase
FIT	fisetin
EE	encapsulation efficiency
LC	loading capacity
